# Comparison of conventional, burst and high-frequency spinal cord stimulation on pain relief in refractory failed back surgery syndrome patients: study protocol for a prospective randomized double-blinded cross-over trial (MULTIWAVE study)

**DOI:** 10.1186/s13063-020-04587-6

**Published:** 2020-08-03

**Authors:** Maxime Billot, Nicolas Naiditch, Claire Brandet, Bertille Lorgeoux, Sandrine Baron, Amine Ounajim, Manuel Roulaud, Aline Roy-Moreau, Géraldine de Montgazon, Elodie Charrier, Lorraine Misbert, Benjamin Maillard, Tanguy Vendeuvre, Philippe Rigoard

**Affiliations:** 1 PRISMATICS Lab (Predictive Research in Spine/Neuromodulation Management and Thoracic Innovation/Cardiac Surgery), Poitiers University Hospital, Poitiers, France; 2 Pain Clinic, Nord-Deux-Sèvres Hospital Center, Thouars, France; 3 Pain Clinic/Palliative Care, Hospital Center La Rochelle, La Rochelle, France; 4 Pain Management and Research Centre, Poitiers University School of Medicine, Poitiers, France; 5 Spine and Neuromodulation Functional Unit, Poitiers University Hospital, Poitiers, France; 6 Institut Pprime UPR 3346, CNRS, ISAE-ENSMA, University of Poitiers, Poitiers, France; 7 Department of Orthopaedic Surgery and Traumatology, Poitiers University Hospital, Poitiers, France; 8 ABS Lab, University of Poitiers, Poitiers, France

**Keywords:** Failed back surgery syndrome, “Paraesthesia free” spinal cord stimulation, Sub-paraesthestic stimulation, Randomized controlled trial, Back pain, Waveform, Chronic pain

## Abstract

**Background:**

While the evolution of technology provides new opportunities to manage chronic refractory pain using different waveform modalities of spinal cord stimulation in failed back surgery syndrome (FBSS), there is no randomized controlled trial available to compare the efficacy of these different stimulations waveforms to date. MULTIWAVE is a prospective, randomized, double-blinded, crossover trial study designed to compare the clinical efficacy of tonic conventional stimulation (TCS), burst stimulation (BURST) and high-frequency stimulation (HF) in FBSS patients over a 15-month period in SCS implanted patients.

**Methods/design:**

Twenty-eight patients will be recruited in the Poitiers University Hospital, in Niort and La Rochelle Hospitals in France. Eligible patients with post-operative low back and leg pain with an average visual analog scale (VAS) score ≥ 5 for low back pain are implanted and randomly assigned to one of the six arms (in a 1:1:1:1:1:1 ratio), where they receive a 3-month combination of TCS, BURST and HF including one treatment modality per month and varying the order of the modality received within the six possible combinations. Patients receiving intrathecal drug delivery, peripheral nerve stimulation and back resurgery related to the original back pain complaint and experimental therapies are excluded from this study. Patients included in the spinal cord stimulation group undergo trial stimulation, and they all receive a TCS treatment for 2 months, as the gold standard modality. Thereafter, patients are randomly assigned to one of the six arms for the total duration of 3-month crossover period. Then, patients choose their preferred stimulation modality (TCS, BURST, or HF) for the follow-up period of 12 months. Outcome assessments are performed at baseline (first implant), before randomization (2 months after baseline) and at 1, 2, 3, 6, 9 and 15 months post-randomization. Our primary outcome is the average global VAS of pain over 5-day pain diary period between baseline and after each period of stimulation. Additional outcomes include changes in leg and back pain intensity, functional disability, quality of life, psychological state, paraesthesia intensity perception, patient satisfaction and the number of adverse events.

**Discussion:**

Recruitment began in February 2017 and will continue through 2019.

**Trial registration:**

Clinicaltrials.gov NCT03014583. Registered on 9 January 2017.

## Background

Failed back surgery syndrome (FBSS), defined by chronic back and/or leg pain after lumbosacral spine surgery [1–5], is reported for 5–55% of the patients post-operatively [6]. FBSS is characterized by mixed neuropathic and nociceptive pain components described as refractory. Pain is defined as refractory, regardless of aetiology, when (i) multiple evidence-based biomedical therapies used in a clinically appropriate and acceptable fashion have failed to reach treatment goals, which may include adequate pain reduction and/or improvement in daily functioning, or have resulted in intolerable adverse effects, and when (ii) psychiatric disorders and psychosocial factors that could influence pain outcomes have been assessed and appropriately addressed [7]. FBSS can have a dramatic impact on functional ability, social aspects and quality of life, resulting in a considerable financial burden on the society [8].

Studies have clearly demonstrated the clinical efficacy and medico-economic interest of spinal cord stimulation (SCS), which consists in delivering an electrical current to the dorsal epidural space via electrodes, as a way of managing refractory pain in FBSS patients [9, 10]. However, the most widely studied and used technique for SCS remains tonic conventional stimulation (TCS), delivering low frequencies (< 100 Hz), which does not appear to relieve pain in more than 30–55% of FBSS patients [9–11]. Moreover, low-frequency SCS provokes paraesthesia, which can ultimately be perceived by some implanted patients as an uncomfortable sensation [12]. These findings have compelled the industry to investigate new sub-paraesthetic stimulation modalities, such as burst (BURST) and high-frequency (HF) stimulation waveforms [13].

Recent clinical trials provide evidence for the efficacy of BURST [14–17] and HF [18–21] SCS in FBSS patients. The main added values of BURST and HF SCS could lead to the elimination of paraesthesia and have a synergistic effect by targeting different intra-spinal structures, mechanisms of action and brain cortical areas in comparison with TCS [22]. RCT evidence is clearly needed to compare the efficacy of TCS, BURST and HF modalities in FBSS patients implanted with new generation SCS.

### Aims and objectives

The MULTIWAVE study is an RCT comparing clinical SCS efficacy using TCS, BURST and HF modalities in a crossover design in implanted chronic refractory FBSS patients. Before randomization, all implanted patients receive TCS treatment for 2 months. Then, TCS, BURST and HF waveforms are randomly and alternatively administered for a 1-month period and a total duration of 3 months for the three different SCS waveforms. After the 3-month period, each patient selects his or her preferred stimulation modality with a follow-up period of 12 months.

The primary objective of this study is to compare the efficacy of TCS, BURST and HF Stimulation on pain relief (measured using a visual analog scale) in refractory FBSS patients. Secondary objectives are to compare changes in the following outcomes for each of the three treatment modalities: back pain intensity, leg pain intensity, functional disability, quality of life and psychological state.

## Methods/design

This protocol is reported in accordance with the SPIRIT 2013 guidelines for protocols of clinical trials [23].

### Design and setting

**Fig. 1:**
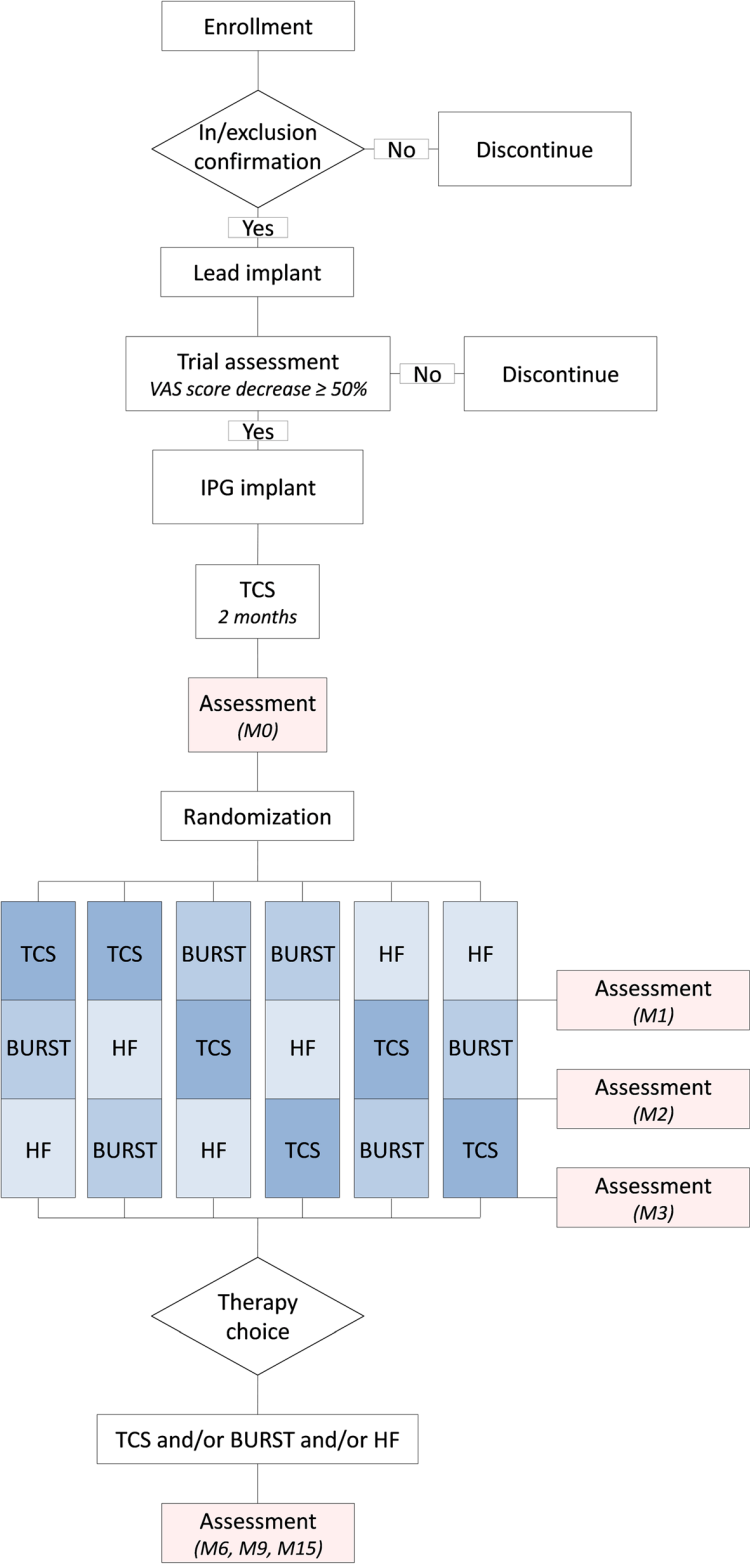
MULTIWAVE trial patient flow

MULTIWAVE study is a prospective, controlled, randomized, crossover, double-blind trial comparing TCS, BURST and HF SCS treatments. Twenty-eight patients will be recruited in the Poitiers University Hospital, in Niort and La Rochelle Hospitals in France. Patients will be implanted with a 32-contact surgical lead and a Precision Spectra SCS System™ (Boston Scientific Inc., Valencia, USA) Internal Pulse Generator (IPG) enabling use of these different waveforms. After a period of 2 months following the surgical lead implant, patients will be randomized 1:1:1:1:1:1 to various SCS modalities. During this period, patients will receive a 3-month specific set of 3 combinations of TCS, BURST and HF amongst 6 possible combinations, delivering each treatment modality during a 1-month period. Patients will be unable to switch from one waveform to another modality until the end of the crossover period. After completion of the 3-month post-randomization period, patients will be asked to choose their preferred SCS modality. Starting with this visit, patients will be able to switch from a waveform to another depending on daily activity within a day. In case the choice of waveform is not obvious, to assist decision making, the patient will be allowed to see his VAS score and pain mapping. Reasons for modality choice will be collected. Patients are monitored up to 15 months post-randomization. The study design is presented in Fig. 1.

### Patient selection

The study population comprises patients suffering from chronic significant low back and leg pain following spinal surgery (so called “FBSS”). A patient must meet all the inclusion criteria and none of the exclusion criteria to be eligible for the study. Inclusion/exclusion criteria will be checked by the Principal Investigator, who is an expert neurosurgeon specialized in surgical SCS implantation.

### Inclusion criteria

The patient is an eligible candidate for SCS, according to the French guidelines for SCS selection and implantation (an average 7-day trial period was completed after lead implantation in all patients assigned to receive SCS). The pain should be refractory despite well-conducted conservative management and no further spine surgery required. For the purposes of this study, FBSS is defined as persistent or recurrent low back and leg pain of at least 6 months’ duration, following at least one decompression and/or fusion procedure [1–5]. The patient has persistent low back and leg pain despite other treatment (pharmacological, surgical, physical, or psychological therapies) that have been tried and did not prove satisfactory, are unsuitable, or are contraindicated for the subject, comprising documented neuropathic characteristics of the radicular and/or low back pain component (Neuropathic pain Diagnostic questionnaire (DN4): sensorimotor testing, clinical examination, pain characteristics, etc.), and presents average back pain ≥ 50 mm over 100 mm, where 0 mm represents no pain and 100 mm the worst imaginable pain, as assessed by the baseline visual analog scale (VAS). Mean daily VAS score calculated on 5 consecutive days.

### Exclusion criteria

The patient is treated or has previously been treated with SCS, subcutaneous or peripheral nerve stimulation, treated with an intrathecal drug delivery system or requires back surgery at the location related to his/her original back pain complaint or receives/had received experimental therapies; had most recent back surgery less than 6 months ago; presents low back pain accessible to etiological “mechanical” surgical treatment (discogenic low back pain, vertebral instability, spinal deformity, etc.); is < 18 years or > 80 years of age; presents a surgical, anaesthetic or psychiatric contraindication to implantation of a SCS system; is pregnant or planning to become pregnant during the course of the study; would be unable to operate the SCS equipment, based on the opinion of the principal or sub-investigator; and is a member of a vulnerable population.

A patient is considered enrolled in the study upon completion of the informed consent process. The subject records his/her global, back and leg pain scores during a 5-day pain diary period prior to randomization. FBSS diagnosis and candidacy for SCS is confirmed based on appropriate imaging according to usual practice. In addition, in cases where psychological and anaesthetic evaluations are standard of care and/or required, the evaluations must take place prior to randomization.

### Interventions

**Fig. 2:**
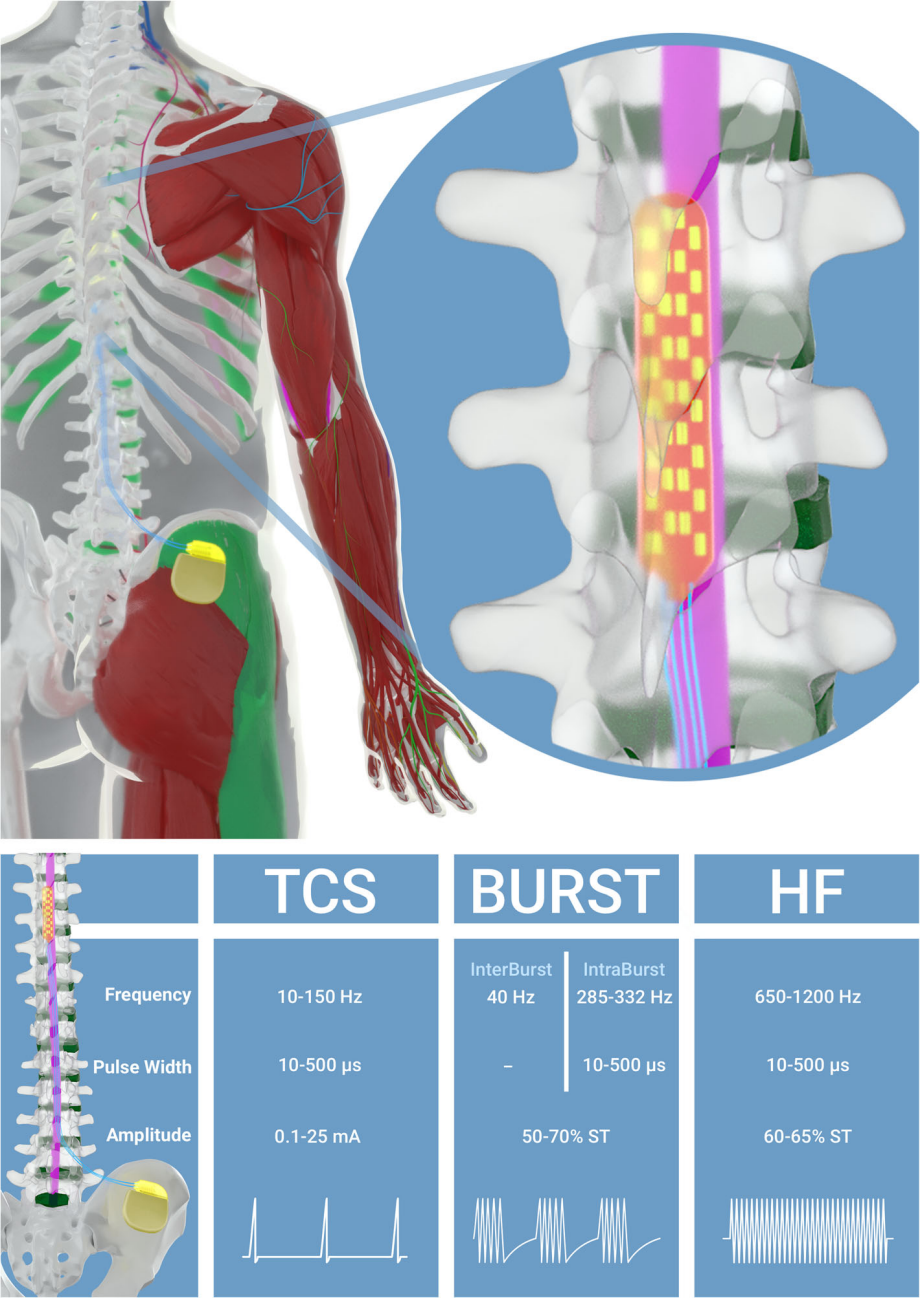
An artistic view illustrating SCS implantation with a 32-contact surgical lead. TCS, BURST and HF waveforms are described by frequency, pulse width and amplitude

Subjects who meet the inclusion criteria and none of the exclusion criteria undergo a 7-day SCS screening trial period with a CoverEdge 32-contact surgical lead™ implantation (Fig. 2).

The implantation procedure describes the various steps of the surgery until final implantation of the 32-contact surgical lead. Procedure length is about 60 min.

#### Phase 1: 32-contact surgical lead insertion

For this procedure, in order to optimize patient cooperation, patients will be placed in prone position and implanted under Target controlled IntraVenous Anaesthesia (TCIVA) allowing patient feedback or general anaesthesia. A CoverEdge 32-contact surgical lead will be implanted empirically at T8/T9 vertebral level, using a minimally invasive technique according to our usual practice. The projection of the conus medullaris over L1-L2 will be assessed on pre-operative MRI, previously to lead implantation to ensure optimal localization of the surgical lead. Immediate fluoroscopic control of the vertebral projection, the lateralization of the lead and lead impedance check will be performed.

#### Phase 2: Electrophysiological testing and “sweet spot” intra-operative mapping for patients under awake anaesthesia by TCIVA only

After radiological assessment of the lead positioning by X-rays, preliminary lead electrophysiological testing will be performed and the lead will subsequently be navigated longitudinally through the epidural space, to determine the best vertebral level and implantation site, using patient feedback and objective quantitative measurements by tactile interface mapping data (NeuroMapping Tools Software) [24, 25]. The goal of the lead implantation is to address the sweet spot location (an anatomical area located within the dorsal columns (DC) of the spinal cord), able when electrically stimulated to generate significant paraesthesia in the appropriate back dermatoma.

#### Phase 3: 32-contact lead programming testing (for patients under awake anaesthesia by TCIVA only)

Some TCS patterns will be tested using various predetermined combinations, in order to achieve good pain coverage, by targeting the sweet spot location with objective quantitative measurements by tactile interface mapping data (NeuroMapping Tools Software) [24, 25]. This is facilitated by our integrated operating theatre, dedicated to SCS implantation, including several of the new computer-assisted and imaging-assisted technologies (multiple screens with live wall projection, full HD camera filming patient feedback and C-arm screen implementation, HF microphones and earphones enhancing surgeon/patient per-operative interactions).

#### Phase 4: 32-contact surgical lead anchoring

After completion of lead programming testing, the lead will be permanently implanted and secured with appropriate anchoring, at its final vertebral level. A final X-ray check will be performed to ensure that the lead has been adequately secured and will not be subject to displacement, once the lead programming and the patient coverage are optimal [24, 25].

#### Phase 5: IPG implantation

Subjects, who succeed in the TCS SCS screening test (global pain VAS score decrease ≥ 50% after a 7-day period trial according to the French Health Authority guidelines), receive a Precision Spectra SCS System™ IPG. At this stage, subjects receive TCS during 2 months before the randomization to avoid any period-related bias due to the pain scar. After the 2 -month period, the subject is randomized to one of the six arms. After the crossover period, subjects choose their preferred modality for a follow-up period of 12 months. Subjects who fail the screening test are excluded from this study.

### Spinal cord stimulation programming modalities

#### Paraesthesia-based waveform using Illumina 3D™ Algorithm

Supported by Multiple Independent Current Control (MICC) hardware, Illumina3D™ is a model-based anatomically guided 3D Neural targeting programming algorithm, designed to precisely calculate anodic and cathodic current distribution at each lead contact. The resulting field shape (or Central Point of Stimulation) is then used as the target for the delivery of various waveforms, including paraesthesia-based stimulation waveform, BURST and HF using Illumina3D™ Algorithm (Fig. 2).

#### Tonic conventional stimulation

TCS patterns are tested thanks to patient cooperation, using various predetermined combinations, in order to achieve good pain coverage and validate the sweet spot location. TCS operates at frequency range of 10–100 Hz for every patient with pulse width of 10–500 μs and amplitude 0.1–25 mA. TCS significantly increases the spontaneous activity of neurons in the gracile nucleus, which are known to project to the primary somatosensory cortex in the brain. This increased spontaneous activation of the spinal nucleus may account for the sensation of paraesthesia that typically occurs during TCS [26].

#### Burst

BURST stimulation consists of closely spaced, high-frequency stimuli delivered to the spinal cord. The stimulus paradigm consists of a 40-Hz burst mode of constant-current stimuli with 5 spikes at 285–332 Hz per burst, pulse width of 10–500 μs, interspike intervals of 3 ms and amplitude corresponding to 50–70% of the sensitive threshold. The differences between TCS and BURST stimulations could be due to more selective modulation of the medial pain pathways by BURST stimulation, as evidenced by activation of the dorsal anterior cingulated cortex [27]. Recent literature supplies good evidence for the efficacy of BURST SCS in the treatment of FBSS with chronic neuropathic radiculopathy [14, 15, 17].

#### High frequency

While HF is generally operated with a range of 500 Hz to 10 kHz, new SCS systems have been developed and make it possible to deliver stimulation up to 10 kHz. The device allows delivery of HF stimulation from 650 to 1200 Hz with pulse width of 10–500 μs, and amplitude corresponding to 60–65% of the sensitive threshold. HF may offer several distinct benefits over TCS. The first would suggest that this therapy could address axial back pain more effectively, which often does not respond as well to traditional SCS. The second is based on the fact that HF, as BURST SCS, appears capable of delivering pain relief without inducing any paraesthesia. This last observation could have obvious benefits for patient acceptance of SCS therapy and could also simplify system implantation by avoiding intraoperative paraesthesia mapping, required to ensure proper lead positioning [28]. The efficacy of HF SCS is still debated in the literature [18, 19, 29, 30].

### Clinical assessments

Outcome measures selected for this trial are based on a review of the previous RCTs of SCS and consideration of IMMPACT recommendations [31]. The visit of randomization is M0 for the study period. Subjects are assessed prior to randomization (M0) and at 1-month, 2-month, 3-month, 6-month, 9-month and 15-month follow-up visits. Assessments are performed by appropriately trained and delegated study staff. The summary of data collection is presented in Table 1.

**Table 1:** Multiwave study—summary of data collection

Study procedure	Enrollment visit	Implantation visit (inclusion)	M_0_ visit (randomization)	M_1_	M_2_	M_3_	M_6_	M_9_	M_15_
Patient information	x								
Informed consent form	x								
Inclusion and exclusion criteria	x	x							
Thoracolumbar X-rays	x	x							
Medical and surgical history (including TENS therapy)	x								
Analgesic treatments, concomitant medications and non-drug treatments	x	x	x	x	x	x	x	x	x
DN4 questionnaire (neuropathic pain)	x								
N3MT (NeuroMapping Tool) pain surface, intensities, paraesthesia coverage	x	x (pre/per/post-implantation)	x	x	x	x	x	x	x
Paraesthesia perception VAS		x (pre/post-implantation)	x	x	x	x	x	x	x
Global, back and leg pain VAS	x	x	x	x	x	x	x	x	x
Patient diary (complete within 1 week prior to visit)		x	x	x	x	x	x	x	x
Nasal *Staphylococcus aureus* decolonization (2 days prior to visit till 2 days after)		x							
Phone call*		x	x	x	x	x	x	x	x
EUROQOL 5-Dimensions (EQ5D)	x	x	x	x	x	x	x	x	x
Oswestry Disability Index (ODI)	x	x	x	x	x	x	x	x	x
Hospital Anxiety and Depression Scale (HADS)	x	x	x	x	x	x	x	x	x
Patient Satisfaction/Patient Global Impression of Change (PGIC)			x	x	x	x	x	x	x
Programming session		x	x	x	x	x	x	x	x
Adverse event/severe adverse event	x	x	x	x	x	x	x	x	x

*Phone call: 1 week prior to the planned visit (patient diary)

### Primary outcome

The primary outcome is the variation of average global VAS score, assessed over a 5-day pain diary, between baseline (randomization, M0) and after each treatment period [31]. Patients record their global, back and leg pain using a (paper) pain diary once a day, for a 5-day period within 1 week prior to each scheduled study visit.

### Secondary outcomes

Secondary outcomes are variation of leg and back pain relief, as measured by VAS [32]; proportion of responders in each group, as responder defined by ≥ 50% reduction of pain; variation of functional disability, as measured by the Oswestry Disability Index Questionnaire (ODI) [33]; variation of quality of life, as measured by EuroQol-5 Dimension (EQ5D) [34]; variation of anxiety and depression, as measured by Hospital Anxiety and Depression Scale (HADS) [35]; variation of paraesthesia, as measured by VAS; the patient satisfaction, as measured by Patient Global Impression of Change (PGIC) [36]; and adverse events, device deficiencies and concomitant treatments.

Further, pain surface, pain intensities and paraesthesia surface are quantified with objective quantitative measurements (Neuro-Pain Software) to compare Global Pain Surface reduction, pain intensity reduction and paraesthesia coverage surface between arms groups [24, 25].

### Process measures

At the screening and baseline visits, the following additional pieces of information are collected: subject demographics (for example, age, gender) and a neuropathic pain assessment by clinician-administered diagnostic questionnaire (e.g. Douleur Neuropathique en 4 questions) to discriminate neuropathic pain components of low back pain [37], surgical and medical history, radiological assessments of thoracolumbar (X-rays) and Nasal *Staphylococcus aureus* decolonization (2 days prior to visit until 2 days after). Subjects who proceed to device implantation have the following information collected: pain/paraesthesia by tactile interface mapping data (NeuroMapping Tools software), lead programming parameter settings, electrode location and device implant information. The NeuroMapping tool is a validated quantitative tactile interface allowing the patient to delineate painful zones in the back and legs and to precisely map objective changes in pain coverage and SCS performance [24, 25].

Unscheduled patient visits could occur between scheduled study follow-up visits due to patient discontinuation from the study or device reprogramming and management of any complication.

### Sample size and power calculations

To calculate the sample size of our study, we started by focusing on the pairwise comparison of HF stimulation and TCS (using a Wilcoxon signed-rank test) while taking into account the Bonferroni correction. BURST and HF stimulation did not show any differences in the literature [21], which was lacking during the development of our study. However, studies comparing HF stimulation and TCS were available, and the paper by Kapural et al. [38] was the largest study comparing these two modalities. In their research, they found a difference of − 1.7/10 95% CI = [− 2.6; − 0.8] points in pain VAS between the two independent groups of HF stimulation and TCS. We decided to proceed with a minimum change of 2.0/10 points as it was found to be the minimum clinically significant difference in pain VAS [32]. To estimate the standard deviation of the difference between treatments while taking into account our crossover design, we needed to estimate intra-subject correlation. It was recommended in the Cochrane handbook to impute missing intra-subject correlation by (SD_1_^2^ + SD_2_^2^ − SD^2^_change_)/(2 × SD_1_ × SD_2_) where SD_1_ = 3.0 and SD_2_ = 2.5 are the standard deviations of the two time points/treatments. Based on data from Kapural et al. [38], we estimated intra-subject correlation of 0.52. We have chosen a correlation of 0.60 as we thought that it would be larger for real paired samples.

Assuming a mean difference of 2 points, a standard deviation of the difference of 2.5 (the square root of SD_1_^2^ + SD_2_^2^ − 2 × corr × SD_1_ × SD_2_ = the square root of 3.0^2^ + 2.5^2^–2 × 0.6 × 3.0 × 2.5), a significance level of 0.0167 (Bonferroni correction) and a power of 0.8, a total sample size of 23 is required and stepped up to 24 in order to ensure a balanced number of subjects per arm. To consider 15% dropout, we decided to include 28 patients.

We also conducted a simulation of 100,000 samples to conduct Friedman tests using the following hypotheses: A difference of 2.0 points between TCS and HF stimulation (5.0/10 (SD = 2.5) vs 3.0/10 (SD = 3.0)), no difference between BURST and HF stimulation (3.0/10 (SD = 3.0) vs 3.0/10 (SD = 3.0)), a correlation of 0.60 between every two treatments (included in the multivariate normal distribution covariance matrix) and a significance level of 0.05. We obtain an estimated power of 96.1% for 28 patients and 90.9% for 23 patients, for detecting the hypothesized difference between the three groups.

### Procedures to minimize bias

To minimize selection bias, randomization sequence is prepared using a random selection programme developed under SAS V9.3. Randomization numbers are assigned in strict sequence, i.e., when a subject is confirmed eligible for randomization, the next unassigned randomization number in the randomization sequence is given. Randomization allocation is hidden from the clinician and subject, using a centralized automatic web-based data management system (https://www.dirc-hugo-online.org/csonline) dedicated to the study and accessible to investigators by username and personal password. Once given, the randomization assignment for the subject cannot be changed. Early departure from the study does not give rise to replacement or reassignment of the rank of inclusion.

Due to the paraesthesia generated by the TCS waveform, the study can be blinded only with HF and BURST stimulation modes. Indeed, for these two types of stimulation, the first step consists in increasing stimulation amplitude until the patient starts feeling paraesthesia. At this amplitude, the programme (combining anodes and cathodes) with the optimal coverage of pain surface is selected. Once the optimal programme is chosen, the minimal threshold allowing paraesthesia perception is identified. Based on it, the patient device remote control is adapted so that patients can modulate stimulation intensity (BURST or HF) only from 20 to 50% of this reference amplitude. By following these recommendations of the manufacturer, patients do not feel paraesthesia with BURST and HF stimulations, which ensures the patient-blinding trial. Double-blind is ensured by conducting patient evaluation and SCS programming by two different agents. Unblinding is made according to the sponsor site procedure. The randomization list is held by the Pharmacy of the CHU of Poitiers.

### Statistical analysis

Categorical variables will be described by numbers and percentages while quantitative variables will be described by the mean and its confidence interval or median and interquartile range based on the skewness of the variable. The study sample will be described by age, gender, BMI, duration between pain appearance and device implantation, number of spinal surgeries and type of pain (neuropathic/mechanical). The primary hypothesis of the difference between TCS, BURST and HF will be verified using the Friedman test or repeated measures ANOVA depending on the normality of the global pain VAS (average VAS over the 5-day diary period). Shapiro-Wilk test will be used to test for non-normality of distributions. In case the Friedman test indicates significance, a post hoc test using the procedure described by Conover [39] and the Bonferroni *p* value adjustment method will be conducted for pairwise comparisons. Ordinal outcomes will be compared between groups using the same previously described procedure. Quantitative normally distributed endpoints will be compared between groups using repeated measures ANOVA. Pairwise comparisons will be conducted after the repeated measures ANOVA indicate significance. Binary outcomes will be compared using the Cochran Q test and a McNemar test will be conducted for pairwise comparison after the Cochran Q test rejects the null hypothesis.

After the end of the crossover design, patients will choose a preferred waveform and will be followed for 1 year. The proportions of waveform preference will be compared using a chi-square goodness of fit test. The categorical variables will be compared between visits M6, M9 and M15 and baseline using the McNemar test while the continuous variables will be compared between baseline and the post crossover visits using the paired *t* test or Wilcoxon signed-rank test depending on the variables’ distribution. All comparisons will be two-sided with a level of significance of 0.05. Missing data will be described. Patients with missing data on the primary outcome will be excluded from the analysis. The blind analysis is aimed to be conducted in per-protocol analysis. A robustness analysis including all available data will be conducted on the primary outcome using a Skillings-Mack Test. R software will be used for statistical analysis.

### Ethics and regulatory aspects

This study received funding in 2016 from Boston Scientific grant programme ISS agreement (Investigator Sponsored Study).

Each patient provides informed consent and approval from the Poitiers University Hospital Ethics Committee (CPP Ouest III), the French National Agency for Medicines and Health Products Safety (ANSM) and the General Data Protection Regulation (GDPR) No. 2016-679 of April 27, 2016.

Prior to site initiation or subsequent involvement in study activities, the sponsor provides study training that is relevant and pertinent to the personnel conducting study activities, including investigator responsibilities and device training (for example, study recommendations for implant procedures and programming, and the requirements of the clinical investigational plan, informed consent process and case report forms).

Monitoring visits are conducted periodically every 4 months. Monitors may work with study personnel to determine and recommend appropriate corrective action(s) and to identify trends within the study.

## Discussion

Throughout a 30-year period, SCS has been developed with TCS modality of stimulation as the main treatment method. In two seminal studies published by North et al. [9] and Kumar et al. [40], TCS appeared to be significantly more efficient to relieve at least 50% of pain rather than reoperation or conventional medical management. However, one limitation of TCS is paraesthesia sensation for the patients. Researchers and industry have consequently developed paraesthesia-free SCS such as BURST and HF. In a short-term duration study, Courtney et al. [17] have investigated the effect of 14-day Burst stimulation in 22 subjects who were previously implanted and were using TCS for at least 90 days. Authors reported that BURST reduced pain from 54.0 mm (± 19.8) to 28.3 mm (± 17.3) after 14 days. Amongst the 22 participants, 20 preferred BURST stimulation in comparison with TCS, notably due to pain relief. Then, this study showed that three participants reported a decrease of at least 50% of paraesthesia, and 16 were free of paraesthesia with BURST stimulation. In a randomized placebo controlled trial, De Ridder et al. [16] compared BURST with TCS and Placebo, in a 3-week period with 1 week by randomly administrated modality, in 15 pain patients (12 FBSS, 1 failed neck surgery, 1 myelopathy, 1 myelomalacia). BURST paraesthesia-free stimulation resulted in greater pain and decrease of attention to pain than TCS and placebo conditions. Some other studies have likewise demonstrated that HF did not induce paraesthesia [18–21, 41]. In a prospective, randomized, controlled study, Kapural et al. [30] demonstrated the non-inferiority and the superiority of HF compared to TCS treatment in 198 patients with both back and leg pain. In this study, the number of patients with a decrease of at least 50% of pain score was greater after a 3-month treatment with HF (~ 84%) than TCS (~ 50%). Authors reported that the superiority of HF over TCS remained present after 12 and 24 months [30, 38]. In a recent crossover study, Duse et al. [42] compared the effect of BURST and 1 kHz HF in 28 FBSS patient previously implanted with TCS for at least 1 year. Authors indicated that BURST and HF programming in a 7-day period did not yield to a superior pain relief than TCS modality. At the final visit, pain relief was however greater for the patients who have chosen BURST or HF than TCS programming modality. Half of the patients preferred TCS modality to feel the tingling sensation, while the other preferred BURST or HF for the higher pain relief. Although BURST and HF might be able to potentiate the effect of TCS to manage pain in both low back or leg pain avoiding paraesthesia sensation, our study is the first to compare the three modalities using one device over a 3-month period. Thanks to its design, the current study will provide evidence of SCS therapy efficacy independently of any paraesthetic effect, if BURST and/or HF modalities appear to work. Since paraesthesia are systematically associated with TCS, previous comparison between TCS and BURST or TCS and HF have been done with placebo-controlled design but without blind modality comparison. Therefore, the results of this study would be expected not to be influenced, to a lesser extent, by paraesthetic sensation.

As indicated in a recent review of Head et al. [43], the choice of stimulation waveform is highly patient-dependent. For instance, Duse et al. [42] reported a 50-50 split between TCS and BURST plus HF waveform type. The device used in this study allows use and selection of TCS, BURST and HF based on patient choice. In the first 3 months after randomization, participants will be assigned to the arm protocol to test all three stimulation modalities independently during 1 month/waveform. Thereafter, participants will be free to choose TCS or BURST or HF depending on day period, their position or their activity. For instance, a patient may select TCS with paraesthesia effect during walking activity and BURST or HF without paraesthesia during sleep. We assume that freedom of modality choice will enhance therapy adherence from patient perspective. Compliance is a priority for both health professionals and patients, to control chronic symptoms, prevent medical crises, maintain financial comfort and increase quality of life [44]. For these reasons, adherence has been called “the key mediator between medical practice and patient outcomes” [45]. Non-compliance imposes a considerable financial burden upon health care systems and may alter clinical therapy outcomes. Although adherence may vary over time in an unpredictable way, interventions that involve monitoring, feedback and informational interventions delivered over multiple sessions are probably effective [46].

The MULTIWAVE study design described herein is not free of limitations.

First, our design presents some limitations, notably for blind comparison between TCS involving paraesthesia effect versus the two other waveforms (BURST and HF), which are paraesthesia-free. However, this design will provide the opportunity to blindly compare BURST and HF for the first time.

Second, the crossover design in comparison with a parallel-arm design does not allow us to determine the long-term efficacy of each modality. While we will be able to document the use of each modality between the 3- and 12-month follow-up periods, long-term efficacy will be determined in a non-controlled design. This limitation could nonetheless be considered as a plus-value for the patient, who will be able to use the whole potential of the SCS with modality choice within a day, while adherence of the treatment is reinforced.

Third, it is not possible to guarantee/claim that each stimulation modality might impact, significantly or not, on the outcomes of the modalities used during the randomization phase. A 1-month wash-out period might be relevant to solve this problem, and we strongly believe that it will allow us to avoid any contamination effect. We agree that the feasibility of a wash-out design is challenging and our study design is not intended for this purpose.

This study represents the first randomized controlled, crossover, double-blind trial comparing TCS, BURST and HF treatments. Beyond any waveform comparison, the ability of this new generation SCS system to independently or simultaneously deliver the three SCS modalities to the patients depending on their needs, in real time, should enhance adherence to the treatment. In the future, the concept of therapy choice within a day can be transposed to cylindrical leads enhanced by the multisource concept. Further studies are also needed to determine the underlying physiological mechanisms for each SCS waveform modalities according to clinical outcomes.

## Trial status

The MULTIWAVE trial began patient recruitment in February 2017 and recruitment was completed in December 2019. Follow-up measurements will be closed in May 2021.

## Data Availability

Not applicable.

## Abbreviations

ANSM: French National Agency for Medicines and Health Products Safety
DN4: Neuropathic pain Diagnostic questionnaire
EQ5D: EUROQOL 5-Dimensions
FBSS: Failed Back Surgery Syndrome
GDPR: General Data Protection Regulation
HADS: Hospital Anxiety and Depression Scale
HF: High frequency
IPG: Internal Pulse Generator
MICC: Multiple Independent Current Control
ODI: Oswestry Disability Index
PGIC: Patient Global Impression of Change
SCS: Spinal cord stimulation
TCIVA: Target-Controlled IntraVenous Anaesthesia
TCS: Tonic conventional stimulation
VAS: Visual analog scale

## References

[1] Chan C, Peng P (2011). Failed back surgery syndrome. Pain Med.

[2] Thomson S (2013). Failed back surgery syndrome - definition, epidemiology and demographics. Br J Pain.

[3] Al Kaisy A, Pang D, Desai MJ, Pries P, North R, Taylor RS (2015). Failed back surgery syndrome: who has failed?. Neurochirurgie.

[4] Gatzinsky K, Eldabe S, Deneuville J-P, Duyvendak W, Naiditch N, Van Buyten J-P (2019). Optimizing the management and outcomes of failed Back surgery syndrome: a proposal of a standardized multidisciplinary team care pathway. Pain Res Manag.

[5] Rigoard P, Gatzinsky K, Deneuville J-P, Duyvendak W, Naiditch N, Van Buyten J-P (2019). Optimizing the management and outcomes of failed back surgery syndrome: a consensus statement on definition and outlines for patient assessment. Pain Res Manag.

[6] Macrae WA (2001). Chronic pain after surgery. Br J Anaesth.

[7] Deer TR, Caraway DL, Wallace MS (2014). A definition of refractory pain to help determine suitability for device implantation. Neuromodulation.

[8] Breivik H, Eisenberg E, O’Brien T, OPENMinds (2013). The individual and societal burden of chronic pain in Europe: the case for strategic prioritisation and action to improve knowledge and availability of appropriate care. BMC Public Health.

[9] North RB, Kidd DH, Farrokhi F, Piantadosi SA (2005). Spinal cord stimulation versus repeated lumbosacral spine surgery for chronic pain: a randomized, controlled trial. Neurosurgery.

[10] Kumar K, North R, Taylor R, Sculpher M, den Abeele CV, Gehring M (2005). Spinal cord stimulation vs. conventional medical management: a prospective, randomized, controlled, multicenter study of patients with failed back surgery syndrome (PROCESS study). Neuromodulation.

[11] Shealy CN, Mortimer JT, Reswick JB (1967). Electrical inhibition of pain by stimulation of the dorsal columns: preliminary clinical report. Anesth Analg.

[12] Verrills P, Sinclair C, Barnard A (2016). A review of spinal cord stimulation systems for chronic pain. J Pain Res.

[13] Viswanath O, Urits I, Bouley E, Peck JM, Thompson W, Kaye AD (2019). Evolving spinal cord stimulation technologies and clinical implications in chronic pain management. Curr Pain Headache Rep.

[14] de Vos CC, Bom MJ, Vanneste S, Lenders MWPM, de Ridder D (2014). Burst spinal cord stimulation evaluated in patients with failed back surgery syndrome and painful diabetic neuropathy. Neuromodulation.

[15] De Ridder D, Vanneste S, Plazier M, van der Loo E, Menovsky T (2010). Burst spinal cord stimulation: toward paresthesia-free pain suppression. Neurosurgery.

[16] De Ridder D, Plazier M, Kamerling N, Menovsky T, Vanneste S (2013). Burst spinal cord stimulation for limb and back pain. World Neurosurg.

[17] Courtney P, Espinet A, Mitchell B, Russo M, Muir A, Verrills P (2015). Improved pain relief with burst spinal cord stimulation for two weeks in patients using tonic stimulation: results from a small clinical study. Neuromodulation.

[18] Russo M, Van Buyten J-P (2015). 10-kHz high-frequency SCS therapy: a clinical summary. Pain Med.

[19] Al-Kaisy A, Van Buyten J-P, Smet I, Palmisani S, Pang D, Smith T (2014). Sustained effectiveness of 10 kHz high-frequency spinal cord stimulation for patients with chronic, low back pain: 24-month results of a prospective multicenter study. Pain Med.

[20] Chakravarthy K, Richter H, Christo PJ, Williams K, Guan Y (2018). Spinal cord stimulation for treating chronic pain: reviewing preclinical and clinical data on Paresthesia-free high-frequency therapy. Neuromodulation.

[21] Kinfe TM, Pintea B, Link C, Roeske S, Güresir E, Güresir Á (2016). High frequency (10 kHz) or burst spinal cord stimulation in failed Back surgery syndrome patients with predominant back pain: preliminary data from a prospective observational study. Neuromodulation.

[22] Roy LA, Gunasingha RMKD, Rauck R (2016). New modalities of neurostimulation: high frequency and dorsal root ganglion. Curr Opin Anaesthesiol.

[23] Chan A-W, Tetzlaff JM, Gøtzsche PC, Altman DG, Mann H, Berlin JA (2013). SPIRIT 2013 explanation and elaboration: guidance for protocols of clinical trials. BMJ.

[24] Guetarni F, Rigoard P (2015). The “neuro-mapping locator” software. A real-time intraoperative objective paraesthesia mapping tool to evaluate paraesthesia coverage of the painful zone in patients undergoing spinal cord stimulation lead implantation. Neurochirurgie.

[25] Rigoard P, Nivole K, Blouin P, Monlezun O, Roulaud M, Lorgeoux B (2015). A novel, objective, quantitative method of evaluation of the back pain component using comparative computerized multi-parametric tactile mapping before/after spinal cord stimulation and database analysis: the “Neuro-Pain’t” software. Neurochirurgie.

[26] Tang R, Martinez M, Goodman-Keiser M, Farber JP, Qin C, Foreman RD (2014). Comparison of burst and tonic spinal cord stimulation on spinal neural processing in an animal model. Neuromodulation.

[27] De Ridder D, Vanneste S (2016). Burst and tonic spinal cord stimulation: different and common brain mechanisms. Neuromodulation.

[28] Vallejo R (2012). High-frequency spinal cord stimulation: an emerging treatment option for patients with chronic pain. Tech Reg Anesth Pain Manag.

[29] Perruchoud C, Eldabe S, Batterham AM, Madzinga G, Brookes M, Durrer A (2013). Analgesic efficacy of high-frequency spinal cord stimulation: a randomized double-blind placebo-controlled study. Neuromodulation.

[30] Kapural L, Yu C, Doust MW, Gliner BE, Vallejo R, Sitzman BT (2015). Novel 10-kHz high-frequency therapy (HF10 therapy) is superior to traditional low-frequency spinal cord stimulation for the treatment of chronic back and leg pain: the SENZA-RCT randomized controlled trial. Anesthesiology.

[31] Dworkin RH, Turk DC, Farrar JT, Haythornthwaite JA, Jensen MP, Katz NP (2005). Core outcome measures for chronic pain clinical trials: IMMPACT recommendations. Pain.

[32] Dworkin RH, Turk DC, Wyrwich KW, Beaton D, Cleeland CS, Farrar JT (2008). Interpreting the clinical importance of treatment outcomes in chronic pain clinical trials: IMMPACT recommendations. J Pain.

[33] Fairbank JC, Pynsent PB (2000). The Oswestry Disability Index. Spine.

[34] Herdman M, Gudex C, Lloyd A, Janssen M, Kind P, Parkin D (2011). Development and preliminary testing of the new five-level version of EQ-5D (EQ-5D-5L). Qual Life Res.

[35] Zigmond AS, Snaith RP (1983). The hospital anxiety and depression scale. Acta Psychiatr Scand.

[36] Hurst H, Bolton J (2004). Assessing the clinical significance of change scores recorded on subjective outcome measures. J Manip Physiol Ther.

[37] Bouhassira D, Attal N, Alchaar H, Boureau F, Brochet B, Bruxelle J (2005). Comparison of pain syndromes associated with nervous or somatic lesions and development of a new neuropathic pain diagnostic questionnaire (DN4). Pain.

[38] Kapural L, Yu C, Doust MW, Gliner BE, Vallejo R, Sitzman BT (2016). Comparison of 10-kHz high-frequency and traditional low-frequency spinal cord stimulation for the treatment of chronic back and leg pain: 24-month results from a multicenter, randomized, controlled pivotal trial. Neurosurgery.

[39] Conover WJ (1998). Practical nonparametric statistics.

[40] Kumar K, Taylor RS, Jacques L, Eldabe S, Meglio M, Molet J (2008). The effects of spinal cord stimulation in neuropathic pain are sustained a 24-month follow-up of the prospective randomized controlled multicenter trial of the effectiveness of spinal cord stimulation. Neurosurgery.

[41] Al-Kaisy A, Palmisani S, Smith T, Harris S, Pang D (2015). The use of 10-kilohertz spinal cord stimulation in a cohort of patients with chronic neuropathic limb pain refractory to medical management. Neuromodulation.

[42] Duse G, Reverberi C, Dario A (2019). Effects of multiple waveforms on patient preferences and clinical outcomes in patients treated with spinal cord stimulation for leg and/or back pain. Neuromodulation.

[43] Head J, Jacob M, Sabourin V, Turpin J, Hoelscher C, Wu C, et al. Waves of pain relief: a systematic review of clinical trials in spinal cord stimulationwaveforms for the treatment of chronic neuropathic low-back and leg pain. World Neurosurg. 2019;131:264-274.e3. 10.1016/j.wneu.2019.07.167 31369885

[44] Vermeire E, Hearnshaw H, Van Royen P, Denekens J (2001). Patient adherence to treatment: three decades of research. A comprehensive review. J Clin Pharm Ther.

[45] Kravitz RL, Melnikow J (2004). Medical adherence research: time for a change in direction?. Med Care.

[46] Kripalani S, Yao X, Haynes RB (2007). Interventions to enhance medication adherence in chronic medical conditions: a systematic review. Arch Intern Med.

